# Posttranscriptional Regulation of *RhBRC1* (*Rosa hybrida BRANCHED1*) in Response to Sugars is Mediated via its Own 3′ Untranslated Region, with a Potential Role of RhPUF4 (Pumilio RNA-Binding Protein Family)

**DOI:** 10.3390/ijms20153808

**Published:** 2019-08-04

**Authors:** Ming Wang, Laurent Ogé, Linda Voisine, Maria-Dolores Perez-Garcia, Julien Jeauffre, Laurence Hibrand Saint-Oyant, Philippe Grappin, Latifa Hamama, Soulaiman Sakr

**Affiliations:** IRHS, Agrocampus-Ouest, INRA, Université d’Angers, SFR 4207 QUASAV, 49000 Angers, France

**Keywords:** shoot branching, *RhBRC1*, 3′UTR, PUF protein, sucrose metabolism

## Abstract

The shoot branching pattern is a determining phenotypic trait throughout plant development. During shoot branching, *BRANCHED1* (*BRC1*) plays a master regulator role in bud outgrowth, and its transcript levels are regulated by various exogenous and endogenous factors. *RhBRC1* (the homologous gene of *BRC1* in *Rosa hybrida*) is a main branching regulator whose posttranscriptional regulation in response to sugar was investigated through its 3′UTR. Transformed *Rosa* calluses containing a construction composed of the CaMV35S promoter, the green fluorescent protein (GFP) reporter gene, and the 3′UTR of *RhBRC1* (P35S:GFP::3′UTR*_RhBRC1_*) were obtained and treated with various combinations of sugars and with sugar metabolism effectors. The results showed a major role of the 3′UTR of *RhBRC1* in response to sugars, involving glycolysis/the tricarboxylic acid cycle (TCA) and the oxidative pentose phosphate pathway (OPPP). In *Rosa* vegetative buds, sequence analysis of the *RhBRC1* 3′UTR identified six binding motifs specific to the Pumilio/FBF RNA-binding protein family (PUF) and probably involved in posttranscriptional regulation. *RhPUF4* was highly expressed in the buds of decapitated plants and in response to sugar availability in in-vitro-cultured buds. *RhPUF4* was found to be close to *AtPUM2*, which encodes an *Arabidopsis* PUF protein. In addition, sugar-dependent upregulation of *RhPUF4* was also found in *Rosa* calluses. *RhPUF4* expression was especially dependent on the OPPP, supporting its role in OPPP-dependent posttranscriptional regulation of *RhBRC1*. These findings indicate that the 3′UTR sequence could be an important target in the molecular regulatory network of *RhBRC1* and pave the way for investigating new aspects of *RhBRC1* regulation.

## 1. Introduction

Throughout their life cycle, plants have to continually adjust to the various environmental conditions in which they are growing. The regulation of shoot branching is one important strategy among others to preserve plant survival and optimize the yield potential of agricultural, horticultural, and forestry crops [1,2]. Shoot branching involves a complex regulatory network based on systemic and local interactions of many endogenous and exogenous cues that converge into the bud to modulate its ability to remain dormant or to grow into a new shoot [3,4,5]. *Teosinte branched1* (*TB1*)/*BRANCHED1* (*BRC1*) and its orthologous genes act as integrators of branching signals within axillary buds [6]. Other so-far unidentified master regulators could exist [7].

In monocots, *Teosinte branched1* (*TB1*) from *Zea mays* [8] and its respective homologs in *Oryza sativa* (*OsTB1*) [9] and in *Sorghum bicolor* (*SbTB1*) [10] were found to influence tillering. They encode transcription factors containing a TCP domain, a domain composed of around fifty-nine amino acids that allows for nuclear targeting, DNA binding, and protein–protein interactions [11,12,13]. *TB1* and *OsTB1* are mainly expressed in axillary bud meristems, where they promote bud growth arrest [9,14] and their respective knock-out mutants *tb1* and *fine culm* exhibit an over-branching phenotype [8,9,15]. Similarly, *BRANCHED1* (*BRC1*) and *BRANCHED2* (*BRC2*) are closely related to *TB1* and regulate the branching process in *Arabidopsis* [16]. *BRC1* expression patterns are mostly restricted to axillary buds, anti-correlated with bud outgrowth, and *brc1* mutant phenotypes are non-pleiotropic and exclusively affect axillary bud development [16]. *BRC1*-like genes have also been identified in other plant species [5].

Sugar-dependent bud growth promotion has been reported in many species including peach [16], walnut tree [17], *Rosa* sp. [18,19], and sorghum [20,21,22]. Exogenous supply of sugars was also found necessary to sustain bud outgrowth of one-node cuttings [19,23,24] and *in planta* [25,26], while plant defoliation impaired bud growth [20,21]. Mason et al. (2014) showed that apical dominance strongly correlated with sugar allocation to axillary buds in intact plants, revealing that apical dominance is predominantly maintained by the intense demand of the shoot tip for sugars, and exogenous sucrose supply through the cut petiole mimics plant decapitation and stimulates bud outgrowth [25]. Sucrose could act as a signaling entity because some non-metabolizable sucrose analogs, including lactulose, can trigger bud outgrowth [23,27], probably via the trehalose-6-phosphate pathway in pea [24]. Despite these findings, our knowledge is very limited regarding the molecular bases of sugar-dependent bud outgrowth promotion. The only available data suggest that sugar might be a central component of the branching regulatory network, since sucrose negatively regulates the expression level of *BRC1* [3,25,27]. Kebrom and Mullet (2015) demonstrated that small changes in the photosynthetic leaf area positively affected the expression of *TB1* and consequently the propensity of tiller buds for outgrowth [28]. Using one-node cuttings of *Rosa* sp., Barbier et al. (2015) demonstrated that sucrose-dependent bud outgrowth stimulation could be linked to down- and up-regulation of strigolactone (SL, a branching-repressor hormone) signaling genes and to cytokinin (CK, a branching-inducer hormone) synthesis, respectively [27]. CK and SL are two secondary messengers antagonistically controlled by polarized auxin transport in the stem [29,30], and partly integrated in the bud by the transcription factor BRC1 [3,5,16,31]. 

In plants, sugars also serve as signal molecules and act through an array of signaling pathways including the sucrose, hexokinase, glycolysis/TCA-cycle, and OPPP (oxidative pentose phosphate) pathways [32,33,34,35,36]. In this context, sugars regulate the expression of a large number of genes at different levels, including the transcriptional, posttranscriptional, and posttranslational levels [36]. Many regulation processes relying on the 3′UTR sequence are considered as a powerful strategy for many organisms to flexibly adjust their functioning in response to different inputs. In rice, analysis of reporter mRNA half-lives of *αAmy3* (*α-amylase 3*) demonstrated that the entire 3′UTR and the two subdomains each functioned as destabilizing determinants in the turnover of mRNA in response to sugar supply, and this response was assigned to the “UAUAUAUGUA” motif [37,38]. In maize, *Incw1*, which encodes a cell-wall invertase, has two types of transcripts that differ by their 3′UTR length and seemingly act as regulatory sensors of carbon starvation [39]. The 3′UTR may constitute a link between sink metabolism and cellular translation activity in plants, although no specific 3′UTR-related motif has been identified to date. Nicolai et al. (2006) identified 224 mRNAs, most of them posttranscriptionally repressed by sucrose starvation, allowing the cell to quickly respond to a general decrease of its metabolic activity [40]. Diverse RNA-binding proteins, which regulate many aspects of the RNA metabolism, such as RNA splicing, polyadenylation, capping, modification, transport, localization, translation, and stability, are particularly important for successful posttranscriptional regulation [41,42]. The Pumilio/FBF RNA-binding protein family (PUF family) is a large family of RNA-binding proteins found in most eukaryotes, represented in the genomes of model organisms by multigenic families [43]. The PUF family members take part in posttranscriptional control by binding to specific regulatory *cis*-elements of their mRNA targets, and thereby lead to mRNA decay and translational repression [44]. They also act by promoting ribosome stalling and facilitating the recruitment of microRNAs (miRNAs) and chromosomal instability [45,46,47,48]. 

In plants, only few investigations have been led to discover the role of the PUF protein in growth and development. Tam et al. (2010) showed that APUM2, an *Arabidopsis* PUF protein, bound the RNA of *Drosophila Nanos Response Element I* (*NRE1*) 5′-UGUAUAUA-3′ located in its 3′UTR, and that APUM1 to APUM22 could shuttle between the nucleus and the cytoplasm through an exportin1-mediated pathway, while APUM23 and APUM24 were exclusively localized in the nucleus [44]. They also indicated that the PUF protein was involved in many processes in plants, such as the osmotic stress response, sugar signaling, nutrient metabolism, the drought stress response, or abscisic acid (ABA) signaling. Using three-hybrid screening assays, Francischini and Quaggio (2009) showed that among the 25 identified PUF members in *Arabidopsis*, APUM1 to APUM6 could specifically bind to the *Nanos* response element sequence, which is also recognized by *Drosophila* Pumilio proteins [49]. They also identified an APUM-binding consensus sequence, i.e., a UGUR tetranucleotide, which is present in all targets of the PUF family [43]. The “non-canonical” *Arabidopsis* PUM23 (APUM23) binding sequence is ten nucleotides long, contains a 5′-UUGA-3′ core sequence, and preferentially contains a cytosine in nucleotide position 8 [50]. These investigations showed that the consensus PUF-binding motif might be ubiquitous among eukaryotes. 

The objective of the present study was to investigate whether sucrose-mediated downregulation of *RhBRC1* could involve posttranscriptional regulation through its 3′UTR sequence. Sequence analysis of the 3′UTR of *RhBRC1* showed the presence of 6 putative PUF binding core motifs (UGUR); one of them was found in the “UAUAUAUGUA” motif similar to the motif previously reported for the 3′UTR of *α-amylase 3* [37,38]. Then, the responsiveness of the *RhBRC1* 3′UTR to metabolizable sugars, non-metabolizable sugars and the main effectors of glycolysis/the TCA-cycle and the OPPP was investigated using *Rosa* calluses transformed with the P35S:GFP::3′UTR*_RhBRC1_* reporter construct. We demonstrated that the 3′UTR sequence prevailed in sugar-mediated *RhBRC1* regulation. Next, twelve PUF protein members were isolated from the *Rosa chinensis* genomic sequence; among them only *RhPUF4* was highly expressed in the buds of decapitated plants and in sugar-supplied in vitro-cultured buds, indicating that *RhPUF4* RNA accumulation is positively related to sugar-mediated bud outgrowth. *RhPUF4* expression was mainly and positively responsive to signals from the OPPP. *RhPUF4* is quite close to *APUM2*, which is highly expressed in the shoot meristem. Taken together, these results indicate that transcription of *RhBRC1* could occur in response to sucrose via its 3′UTR, partly through OPPP-dependent upregulation of *RhPUF4*. 

## 2. Results

### 2.1. Sucrose and Glucose Influence the Expression of RhBRC1 through Its 3′UTR 

**Figure 1:**
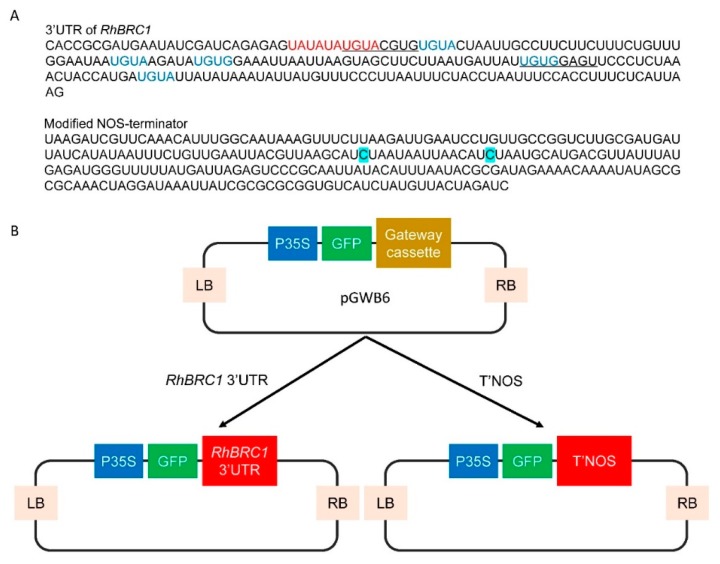
(**A**) Sequences of the *RhBRC1* 3′UTR region and modified NOS-terminator. Red letters, sugar-related motif found in *Oryza sativa α-amylase 3*; *(***B**) schematic diagram of the plasmid used for stable transformation of *Rosa* calluses. Underlined letters, putative APUM2 or RhPUF4 protein binding motifs; blue letters, conserved PUF protein binding core motifs; letters in blue boxes: Modified nucleotide.

**Figure 2:**
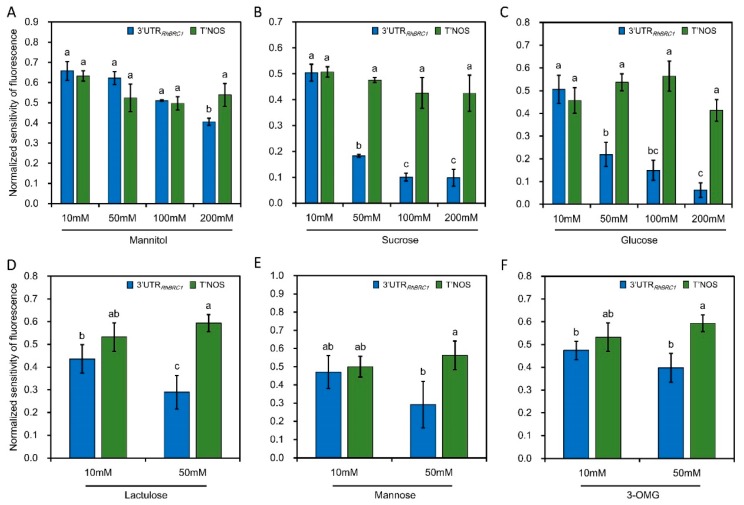
Levels of 3′UTR*_RhBRC1_*-transformed calluses (P35S:GFP::3′UTR*_RhBRC1_*) as compared to T’NOS-transformed calluses (P35S:GFP::T’NOS) following different sugar treatments. (**A**–**C**) Fluorescence levels of 3′UTR*_RhBRC1_*- and T’NOS-transformed calluses treated with different mannitol, sucrose or glucose concentrations, respectively. (**D**–**F**) Fluorescence levels of 3′UTR*_RhBRC1_*- and T’NOS-transformed calluses treated with different lactulose, mannose, and 3-OMG concentrations, respectively. Data are means ± SEs of three measurements, and each measurement was performed on six *Rosa* calluses. The letters indicate significant differences between the different treatments with *p* < 0.05.

Chan and Yu. (1998a,b) showed that the abundance of *α-Amylase 3* in *Oryza sativa* was sugar-repressive and associated with the presence of one of the two UAUAUAUGUA or UAUAUAAUGUA motifs in its 3′UTR [38,51] (Figure S1). Based on the sugar-dependent downregulation of *RhBRC1* [27], we investigated whether its 3′UTR was also involved in this regulation. Its 3′UTR sequence contained the same motif (UAUAUAUGUA) as the one previously reported for *α-Amylase 3* as well as six PUF-binding motifs, while the 3′UTRs of *AtBRC1* and *OsTB1* contain two and four PUF-binding motifs*,* respectively (Figure S1). *Rosa* calluses were thus transformed with a construction composed of the CaMV35S promoter, the green fluorescent protein (GFP) reporter gene upstream of the 3′UTR of *RhBRC1* (P35S:GFP::3′UTR*_RhBRC1_*, Figure 1B). A P35S:GFP::T’NOS construct was also used as a control (Figure 1B). T’NOS is the 3′UTR of the agrobacterial nopaline synthetase gene. For this construct, a modified NOS terminator (T’NOS) without any putative PUF-binding motif was used (Figure 1A). The transformed calluses were first selected based on antibiotic resistance, and the presence of the targeted 3′UTR was confirmed by PCR-mediated DNA amplification. The transformed calluses were then transferred to the incubation medium containing different concentrations of sugars ranging from 10 to 200 mM, and fluorescence intensity was assessed using ImageJ software after 8 h incubation. The 3′UTR*_RhBRC1_*-transformed calluses (P35S:GFP::3′UTR*_RhBRC1_*) were incubated on a medium containing a soluble sugar (sucrose or glucose) for 8 h, and fluorescence strongly decreased as sugar concentrations increased (Figure 2B). Fluorescence was highest in response to 10 mM sucrose (the lowest sugar concentration) and lowest in response to 100 mM sucrose and 200 mM glucose. Incubation on lactulose, a non-metabolizable sucrose analog, decreased GFP intensity (Figure 2D). Meanwhile, the fluorescence level of the control remained almost stable in response to these sugar concentrations, supporting that the 3′UTR*_RhBRC1_* could be a sugar-sensitive sequence (Figure 2B,C). Mannitol (the osmotic control) did not cause any dramatic change in the fluorescence intensity of either the control or the 3′UTR*_RhBRC1_*-transformed calluses, except when its concentration was as high as 200 mM (Figure 2A). These findings support our initial assumption that the 3′UTR sequence of *RhBRC1* could mediate sugar-dependent *RhBRC1* repression through a posttranscriptional process. 

### 2.2. The 3′UTR of RhBRC1 Responds to Glycolysis/TCA-Cycle and OPPP Signaling

**Figure 3:**
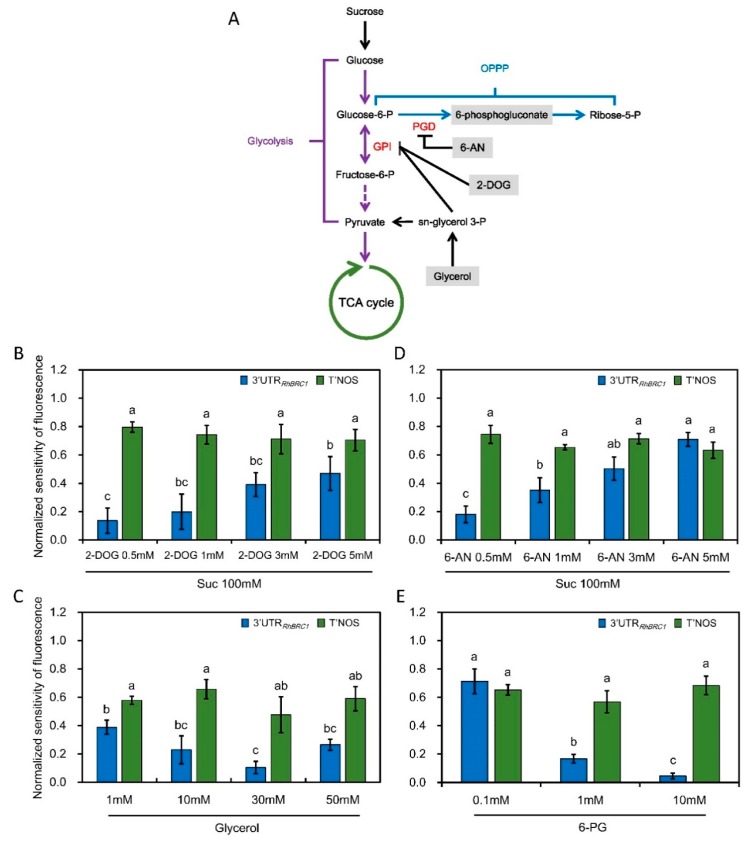
Both glycolysis/the TCA-cycle and the OPPP participate in the posttranscriptional regulation of *RhBRC1* through its 3′UTR. (**A**) Different effectors function at the level of different enzymes in the primary metabolism; (**B**,**D**) fluorescence levels of 3′UTR*_RhBRC1_*- and T’NOS-transformed calluses in response to 100 mM sucrose and different 2-DOG or 6-AN concentrations, respectively; (**C**,**E**) fluorescence levels of 3′UTR*_RhBRC1_*- and T’NOS-transformed calluses in response to different glycerol or 6-AN concentrations, respectively. GPI, glucose-6-phosphate isomerase; GPD, glucose-6-phosphate dehydrogenase; 6-PG, 6-phosphogluconate, Suc, sucrose. Data are means ± SEs of three measurements, and each measurement was performed on six *Rosa* calluses. The letters indicate significant differences between the different treatments with *p* < 0.05.

When the 3′UTR*_RhBRC1_*-transformed calluses were incubated on a medium containing different concentrations of mannose (a slowly metabolizable glucose analog) for 8 h, no significant decrease of fluorescence intensity was observed as the mannose concentration increased (mannose is a marker of the hexokinase dependent pathway) (Figure 2E). Similar results were found with 3-O-methyl-glucose (3-OMG, Figure 2F), a marker of the hexokinase-independent pathway [23]. Hexokinase is an important enzyme that catalyzes the transformation of glucose into glucose-6-phosphate. We then checked whether the downstream hexokinase pathway, glycolysis/the TCA-cycle, and the OPPP were involved in sugar-mediated posttranscriptional regulation of *RhBRC1* through its 3′UTR. The regulation of 3′UTR*_RhBRC1_* callus activity by sucrose metabolism pathways was investigated using 2-deoxyglucose (2-DOG), an inhibitor of glycolysis [52,53], and 6-aminonicotinamide (6-AN), an inhibitor of the OPPP [35,54,55] on sucrose-supplied media [35,56]. Were also tested glycerol that fuels the downstream part of glycolysis while inhibiting glucose-6-phosphate isomerase to form glucose-6-phosphate that is required for the OPPP (Figure 3A), and 6-phosphogluconate that fuels the downstream part of glycolysis and the OPPP (Figure 3A). Sucrose is required to produce glucose and glucose-6-phosphate, the precursors of glycolysis/the TCA-cycle and the OPPP, respectively (Figure 3A). When the 3′UTR *_RhBRC1_*-transformed calluses were co-treated with 100 mM sucrose + 0.5 mM 2-DOG, fluorescence was significantly lower and increased progressively, but not very sharply, to reach its highest level with 100 mM sucrose + 5 mM 2-DOG (Figure 3B). Glycerol treatment confirmed these results: The fluorescence of the 3′UTR*_RhBRC1_*-transformed calluses decreased slightly and significantly to reach its lowest level under 30 mM glycerol, but increased slightly again under 50 mM glycerol (Figure 3C). Under the same experimental conditions, the control transformed calluses exhibited no significant change in fluorescence intensity, supporting a potential role of the 3′UTR in the mediation of glycolysis/TCA-cycle-dependent downregulation of *RhBRC1*. The treatment of the 3′UTR*_RhBRC1_*-transformed calluses with different concentrations of pyruvate, derived from glycolysis, also confirmed this conclusion (Figure S2A).

To determine whether the OPPP could lead to the posttranscriptional regulation of *RhBRC1* through its 3′UTR, 6-AN and 6-phosphogluconate (6-PG) were selected to treat the transformed calluses. When the 3′UTR*_RhBRC1_* calluses were placed on 100 mM sucrose + different concentrations of 6-AN (from 0.5 to 5 mM), fluorescence increased as the 6-AN concentration increased. With the same sucrose concentration (100 mM), the highest fluorescence corresponded to the calluses incubated on 5 mM 6-AN and the lowest one to the calluses incubated on 0.5 mM 6-AN (Figure 3D). The opposite fluorescence pattern was found when 3′UTR*_RhBRC1_* calluses were supplied with 6-PG, a substrate of the OPPP: Fluorescence increased as the 6-PG concentration decreased (Figure 3E). More interestingly, when the calluses were co-treated with 1mM 2-DOG and different concentrations of glucose-6-phosphate (glycolysis/the TCA-cycle are blocked by 2-DOG, and glucose-6-phosphate preferentially fuels the OPPP), the fluorescence level of the 3′UTR*_RhBRC1_* calluses changed significantly, and consistently decreased as the glucose-6-phosphate concentration increased (Figure S2B). Under the same experimental conditions, no significant changes in the fluorescence level of the control-transformed calluses was found, supporting that this regulation was specific to the 3′UTR of *RhBRC1*, which plays a major role in the OPPP-dependent posttranscriptional regulation of *RhBRC1*.

### 2.3. Identification of PUF Family Members in Rosa Chinensis 

**Figure 4:**
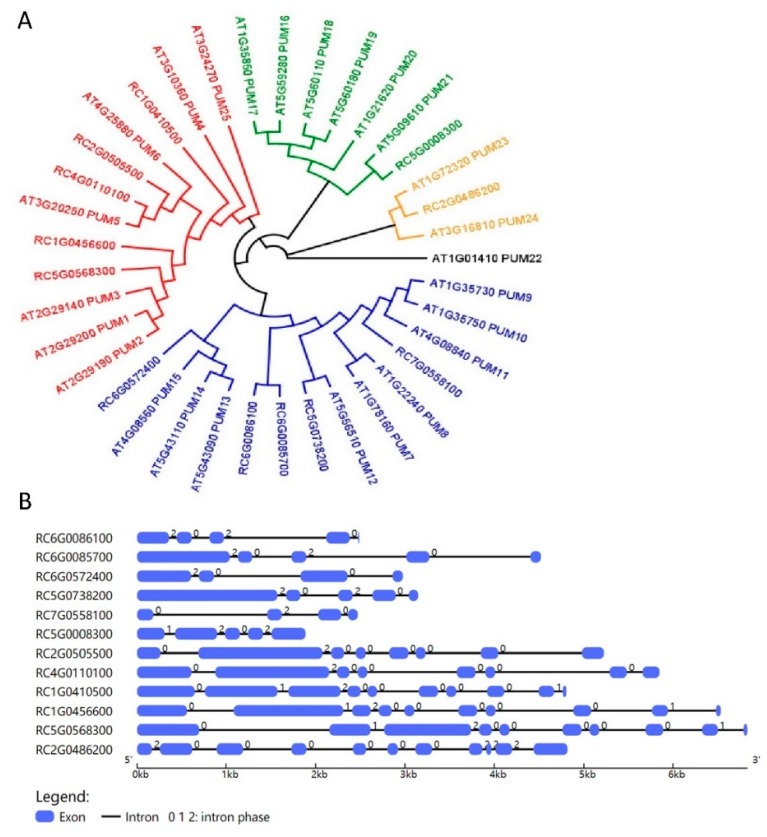
Identification of PUF members in *Rosa chinensis*. (**A**) Phylogenetic tree of PUF members in *Arabidopsis thaliana* and *Rosa chinensis.* The maximum likelihood analysis in the MEGA program was used to build the phylogenetic tree. The PUF sequences of *Rosa chinensis* and *Arabidopsis thaliana* were downloaded from the GDR (https://www.rosaceae.org/) and TAIR (https://www.arabidopsis.org/) databases, respectively. (**B**) Gene structure dynamics of PUF members in *Rosa chinensis*. The gene structures of RcPUF members were obtained using GSDS software (http://gsds.cbi.pku.edu.cn/).

The PUF family is mainly involved in posttranscriptional control by binding to specific regulatory *cis*-elements that contain a UGUR (R: purine) flanked by an AU-rich sequence. Through this interaction, they govern RNA decay and translational repression [44]. Based on the presence of 6 putative PUF-binding core motifs in the 3′UTR of *RhBRC1* (Figure 1A), we hypothesized that PUF proteins might mediate the posttranscriptional regulation of *RhBRC1* in response to sugar. The phylogenetic analysis of the identified *RcPUF* (*Rosa chinensis* PUF) proteins was performed using MEGA7.0 software. According to previous studies, the PUF family includes 26 members in *Arabidopsis,* which can be grouped into five subfamilies through phylogenetic analysis [44] (Figure 4A). In *Rosa chinensis*, we only identified twelve PUF members based on the genome sequence from GDR database [57,58,59], which is far less than in *Arabidopsis*. The PUF protein members can be classified into four groups (Figure 4A), and their gene length varies from 2000 bp to 5000 bp (Figure 4B). Moreover, out of these twelve PUF members, eleven of them contain eight PUF repeats, and only RC7G0558100 contains seven PUF repeats (Figure S3).

### 2.4. The Transcription Level of RhPUF4 Is Regulated by Sugar

**Figure 5:**
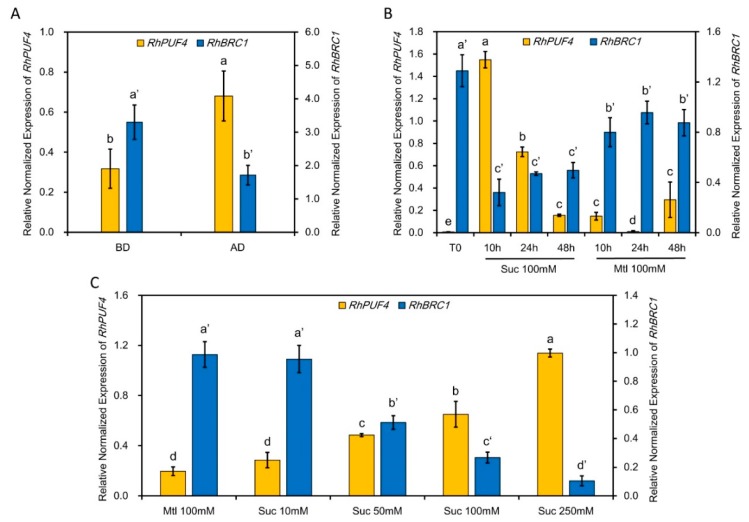
*RhPUF4* expression is under the control of sucrose and the sucrose metabolism, and has an opposite expression pattern to *RhBRC1*. (**A**) Transcription levels of *RhPUF4* and *RhBRC1* in buds before decapitation (BD) or after decapitation (AD); (**B**) transcription levels of *RhPUF4* and *RhBRC1* in *in vitro*-cultured buds after 0 h, 10 h, and 24 h under 100 mM of sucrose or 100 mM mannitol; (**C**) transcription levels of *RhPUF4* and *RhBRC1* in buds treated with different sucrose concentrations. Mtl, mannitol; Suc, sucrose. Data are means ± SEs of three replicates. The letters indicate significant differences between the different treatments with *p* < 0.05.

To check whether PUF family proteins could be involved in sucrose-induced bud outgrowth, the expression patterns of all twelve *RhPUFs* (*Rosa hybrida PUFs*) were investigated by RT-PCR in *in vitro*-cultured buds supplied with 100 mM sucrose (for non-dormant buds) or 100 mM mannitol (for dormant buds) for 24 h. We identified a homologous gene of *Arabidopsis* PUF genes from the complete sequence of *Rosa chinensis,* and we named it *RhPUF*. Using specific primers for each PUF member (Table 1), only *RhPUF4* (RC5G0568300) showed a high expression level in 100 mM sucrose-supplied buds, while hardly any expression was observed in 100 mM mannitol-supplied buds (Figure S4). Furthermore, the expression level of *RhPUF4* increased in a sucrose concentration-dependent manner, supporting the presence of a sugar-inducible gene in growing buds (Figure 5C). Then, we checked the transcription pattern in sucrose- and mannitol-treated calluses. The result confirmed that the transcription level of *RhPUF4* was also dependent on the sucrose concentration, but not on the mannitol concentration (Figure S5A,B). The time course of *RhPUF4* expression in the early stage, prior the onset of rapid bud growth, showed that *RhPUF4* was early (highest level after 10 h) and temporarily expressed in 100 mM sucrose-supplied buds (non-dormant ones), as compared to those supplied with 100 mM mannitol (dormant buds, Figure 5B). In line with this, *RhPUF4* was found more expressed in non-dormant buds (released from apical dominance) than in dormant buds (under apical dominance) (Figure 5A). The *RhPUF4* transcription pattern was inversely correlated with the transcription level of *RhBRC1* in the early stage of bud outgrowth: Its level was lowest in the buds with a high expression level of *RhBRC1* and highest in the buds with a low expression level of *RhBRC1* (Figure 5). Taken together, these findings show that *RhPUF4* expression is early and highly expressed in non-dormant axillary buds, and is negatively correlated with *RhBRC1* expression.

### 2.5. The Transcript Level of RhPUF4 Is More Likely to Be Sensitive to OPPP Signaling 

**Figure 6:**
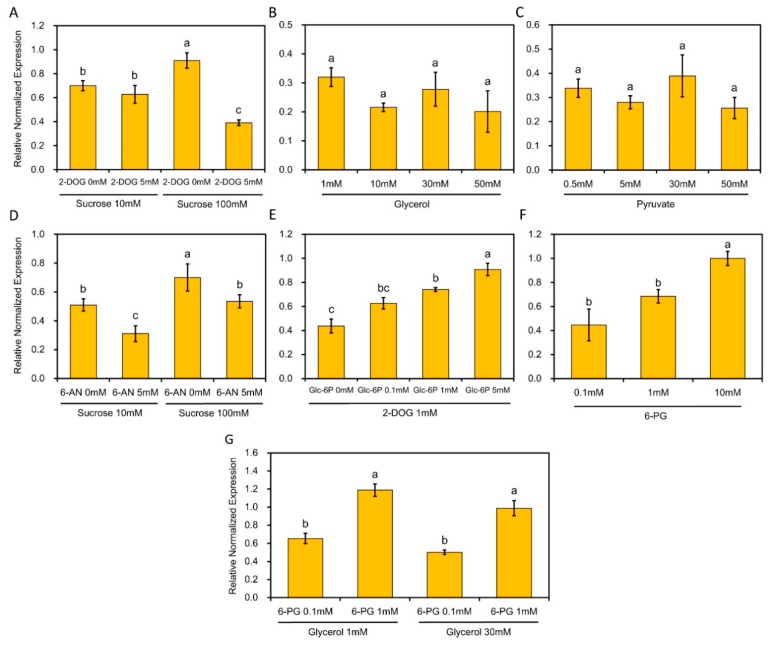
The transcript level of *RhPUF4* is slightly responsive to glycolysis/the TCA-cycle, but highly sensitive to the OPPP in in-vitro-cultured buds. (**A**,**D**) Transcript levels of *RhPUF4* in buds treated with 10 mM and 100 mM sucrose in the presence or in the absence of 5 mM 2-DOG or 6-AN, respectively; (**B**,**C**) transcript levels of *RhPUF4* in buds treated with different concentrations of glycerol or pyruvate, respectively; (**E**) transcript levels of *RhPUF4* in buds treated with 1 mM 2-DOG and different concentrations of glucose-6-phosphate; (**F**) transcript levels of *RhPUF4* in buds treated with different concentrations of 6-PG. (**G**) Transcript levels of *RhPUF4* in buds treated with different combinations of glycerol and 6-PG. Glc-6P, glucose-6-phosphate; 6-PG, 6-phosphogluconate. Data are means ± SEs of three replicates. The letters indicate significant differences between the different treatments with *p* < 0.05.

To further investigate the relationship between *RhPUF4* and sugar-dependent posttranscriptional regulation of *RhBRC1*, we investigated its transcript levels in buds treated with the glycolysis/TCA-cycle effector 2-DOG, as we did for the transformed *Rosa* calluses (Figure 3B). When buds were co-supplied with sucrose and 2-DOG, the *RhPUF4* level did not significantly change under 10 mM sucrose, while it unexpectedly decreased under 100 mM sucrose (Figure 6A). In accordance with this, buds only supplied with glycerol or pyruvate, two compounds in glycolysis/the TCA-cycle, did not exhibit significant changes in *RhPUF4* transcript levels (Figure 6B,C). In order to check whether *RhPUF4* regulation was dependent on OPPP signaling, *RhPUF4* transcription levels were investigated in buds directly in response to OPPP inhibition (sucrose-fed buds supplied with 5 mM 6-AN) or to OPPP activation (buds supplied with 6-PG, a direct substrate of the OPPP). The in-vitro-cultured buds treated with 5 mM 6-AN exhibited higher downregulation of *RhPUF4* under a low (10 mM) than under an elevated (100 mM) sucrose concentration (Figure 6D). Furthermore, 6-PG treated buds displayed a concentration-dependent response of *RhPUF4* transcript levels. The highest level of *RhPUF4* was indeed found when the buds were supplied with 10 mM 6-PG, relatively to 0.1 mM 6-PG (Figure 6F). To confirm this result, the transcript levels of *RhPUF4* were assessed in in-vitro-cultured buds co-treated with 2-DOG (a blocker of glycolysis/the TCA-cycle) and glucose-6-phosphate (used in the OPPP). When 1 mM 2-DOG-supplied buds were supplied with a gradient of concentrations of glucose-6-phosphate (from 0 to 5 mM) to preferentially activate the OPPP, the transcript level of *RhPUF4* increased in a concentration-dependent manner and reached its maximum under 5 mM glucose-6-phosphate (Figure 6E). Moreover, the transcription level of *RhPUF4* in calluses was also more sensitive to the OPPP than to glycolysis (Figure S5C). In addition, exogenous addition of glycerol to 6-PG treated buds did not affect the *RhPUF4* level (Figure 6G), supporting once again that the transcript level of *RhPUF4* could be highly sensitive to the OPPP. 

### 2.6. RhPUF4 Could Bind to the 3′UTR of RhBRC1 and Promote Plant Growth 

**Figure 7:**
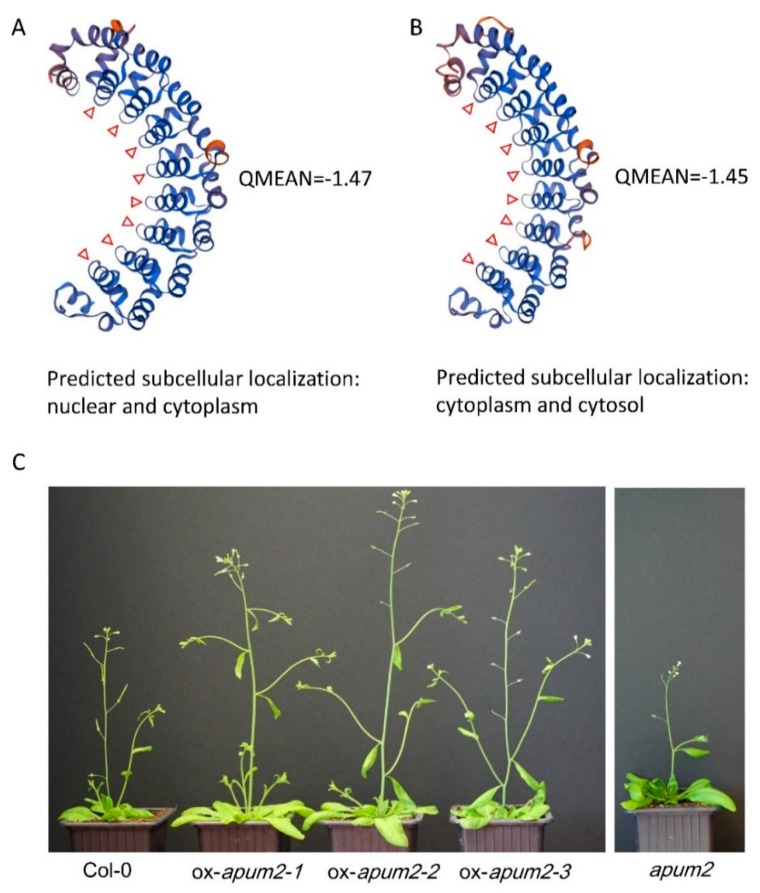
RhPUF4 could promote plant growth and bind to the PUF motifs. (**A**,**B**) Putative tertiary structures of RhPUF4 and APUM2, respectively, based on the prediction of the SWISS-MODEL database (https://swissmodel.expasy.org/); (**C**) phenotypes of ox-APUM overexpression transgenic *Arabidopsis* plants and of the knockout mutant *apum2* as compared to the wild type. The APUM2 and RhPUF4 protein sequences were downloaded from the TAIR (https://www.arabidopsis.org/) and GDR (https://www.rosaceae.org/) databases. The red triangles represent pumilio repeats.

In order to know whether RhPUF4 could bind to the 3′UTR of *RhBRC1*, the NCBI database was used to find the homologous gene of *RhPUF4* in *Arabidopsis*. The BLAST result showed that *APUM2* (*AT2G29190)* was a homologous gene of *RhPUF4,* with a high query cover (99%), a high percent identity (61.76%), and a low E-value (0.0). Moreover, our phylogenetic tree also confirmed that *RhPUF4* and *APUM2* belong to a same cluster (Figure 4A). APUM2 is involved in cell differentiation and highly expressed in the shoot meristem in *Arabidopsis* [60]. It has a high binding affinity to a conserved sequence that contains a core UGUR motif flanked with an NRKR motif [49,50]. Moreover, we also found the UGURNRKD motif in the 3′UTR of *RhBRC1* (Figure 1A). In order to know whether RhPUF4 could also respond to the same motif, we used the SWISS-MODEL database [61,62] to predict the tertiary structure of RhPUF4 and APUM2. The result showed that its structure had a high QMEAN (qualitative model energy analysis) value (−1.47 and −1.45, respectively, Figure 7A,B), and both of them had a conserved eight-pumilio-repeats domain (Figure 7A,B). Furthermore, the WoLF PSORT database was used to predict the subcellular localization of RhPUF4 [63]. This analysis indicated that RhPUF4 was located in the nucleus and the cytoplasm. Both results supported that RhPUF4 and APUM2 could bind to the same motif in the 3′UTR, probably based on their conserved PUF motif. In order to get an insight into the function of RhPUF4 during plant development, we overexpressed its close homolog *APMU2* in *Arabidopsis*. The transgenic *Arabidopsis* plant was more elongated and had a thicker and longer stem than the wild type (Figure 7C). Lastly, the *apum2* knockout mutant exhibited a thinner and shorter stem and no bud outgrowth as compared to the wild type (Figure 7C).

## 3. Discussion

### 3.1. Involvement of the 3′UTR Region in Sugar-Mediated Downregulation of RhBRC1 

*BRC1* and its homologous genes play a central role in shoot branching and are downregulated by sugars [5]. We showed that one of the mechanisms behind the sucrose-dependent downregulation of *RhBRC1* occurred through its 3′UTR sequence, which contains six putative PUF motifs, with one of them present in the reported sugar-related motif (UAUAUAUGUA) (Figure 1A). *BRC1* is regulated at different levels, including the posttranscriptional level, as evidenced by microRNA393-dependent repression of *OsTB1* and stimulation of tillering in rice [64]. Protein interactions also participates in this process: BRANCHED1 interacts with FLOWERING LOCUS T to repress the floral transition of the axillary meristems [65], and TIE1 (TCP interactor containing EAR motif protein 1) can directly interact with BRC1 and repress its binding efficiency [66]. In addition, the expression of some TCP transcription factors belonging to the same family as BRC1 is regulated through posttranscriptional regulation [67,68]. In *Arabidopsis*, miRNA319 can target many TCP transcription factors in response to ABA and CK [69,70]. The 3′UTRs of certain genes are also under SL control. For example, miR156 targets the 3′UTR of the SL-related genes SPL3, SPL9, and SPL15, to regulate shoot branching [71,72,73]. In the present study, exogenous supply of sucrose or glucose indeed decreased the fluorescence of the P35S:GFP::3′UTR*_RhBRC1_*-transformed calluses in a concentration-dependent manner (Figure 2A), while no effect was observed on the control P35S:GFP::T’NOS-transformed calluses (Figure 2A). In line with this, no effect of mannitol was found in either type of transformed callus (Figure 2A). The involvement of the 3′UTR in sugar signaling is limited to cases related to sugar abundance [39,51], and the 3′UTR may constitute a link between the organ metabolism and sugar availability in plants. The exact motif involved in sugar-mediated posttranscriptional regulation is still unknown, but a role of the UAUAUAUGUA sequence has been reported in posttranscriptional sugar-dependent regulation of *α-amylase 3* [38]. Interestingly, this 3′UTR motif exists in *RhBRC1*, supporting that it is conserved between monocots (*Oryza sativa*) and dicots (*Rosa hybrida*). However, it is absent in *AtBRC1* and *OsTB1* (Figure S1). It will be more informative to further test whether *AtBRC1/TB1* could be under sugar-dependent post-transcriptional regulation. 

### 3.2. Posttranscriptional Regulation of RhBRC1 by Sucrose Is Mainly Mediated through the OPPP

The 3′UTR of *RhBRC1* is sensitive to sucrose and lactulose (its non-metabolizable analog), which both induce bud outgrowth and repress *RhBRC1* expression in *Rosa* buds [27]. While a significant decrease occurred in the P35S:GFP::3′UTR*_RhBRC1_* -transformed calluses in response to glucose, no significant decrease occurred in response to mannose, a glucose analog linked to the hexokinase (HXK) signaling pathway [23]. This suggests a minor role of this pathway in the glucose-mediated posttranscriptional regulation of *RhBRC1*. Downstream of HXK, glycolysis/the TCA-cycle and the OPPP are the two most important sugar metabolism pathways. They provide energy for plant development, precursors for amino acid synthesis, and signaling molecules for modulating certain pathways [74,75]. Moreover, they are also involved in the regulation of microRNAs, transcription factors, and in the crosstalk with hormonal, oxidative, and defense signaling [76]. We show here that the two sucrose metabolism pathways (glycolysis/the TCA-cycle and the OPPP) regulate *RhBRC1* abundance at different magnitudes at the posttranscriptional level. The fluorescence level of the P35S:GFP::3′UTR*_RhBRC1_* -transformed calluses indicated that the 3′UTR of *RhBRC1* was slightly but significantly sensitive to glycolysis/the TCA-cycle (Figure 3B,C). Although the sucrose- and 2-DOG- co-treated 3′UTR*_RhBRC1_* calluses displayed increased fluorescence in a 2-DOG-concentration-dependent manner, this difference remained only slightly statistically significant. The same results were also found in the glycerol- and pyruvate-treated 3′UTR*_RhBRC1_*-transformed calluses, indicating that glycolysis and the related TCA-cycle-dependent *RhBRC1* expression could be mildly mediated through its 3′UTR. By contrast to glycolysis, the 3′UTR of *RhBRC1* was found to be significantly responsive to the OPPP (Figure 3D,E). The fluorescence of the 3′UTR*_RhBRC1_*-transformed calluses was indeed activated or inhibited by 6-AN (an OPPP blocker) and 6-PG (an OPPP substrate), respectively. In accordance with this, the combination of 2-DOG (a blocker of glycolysis) and glucose-6-phosphate (preferentially used by the OPPP in the presence of 2-DOG) reduced the fluorescence of the 3′UTR*_RhBRC1_* calluses. All these findings were specific to the 3′UTR*_RhBRC1_* calluses; no significant modification of fluorescence was found in the control transformed calluses. In eukaryote cells, many factors are related to posttranscriptional regulation, such as RNA-binding proteins, microRNAs, protein phosphorylation, or methylation [41,77,78]. Some posttranscription-related mechanisms are believed to be involved in the OPPP. For example, TOR kinase can mediate the upregulation of G6PD (glucose-6-phosphate dehydrogenase, one of key enzymes of the OPPP) and the activity of TOR kinase is probably under the positive regulation of NADPH, a product of the OPPP [79,80]. A recent study showed that TOR kinase could phosphorylate APUM2 in *Arabidopsis* [81]. Moreover, microRNA124 and Hsp27 in *Homo sapiens* have also been reported to be involved in the OPPP [82,83]. To date, the involvement of the OPPP in posttranslational control has not been documented in plants; our results open the way onto this novel mechanism in relation with shoot branching. 

### 3.3. Involvement of RhPUF4 in Posttranscription of RhBRC1 Mediated by the OPPP 

**Figure 8:**
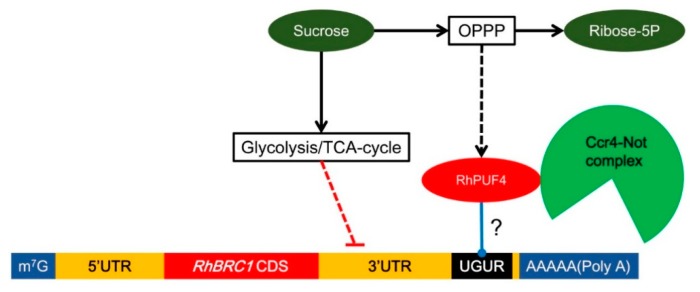
RhPUF4 could bind the 3′UTR of *RhBRC1* to regulate its expression. There exist putative PUF-binding sites in the 3′UTR of *RhBRC1*. APUM2 proteins can recruit the Ccr4-Not complex to the 3′UTR of target mRNAs and enhance the degradation of target mRNAs by cutting the poly(A) tail [89,90,91]. Moreover, the transcription of *RhPUF4* is stimulated by the OPPP, which is itself enhanced by sucrose. Blue line means protein interaction. Dashed line means indirect effect.

The OPPP and glycolysis decreased the expression of *RhBRC1* through its 3′UTR, even if glycolysis had a weak effect (Figure 3). However, the posttranscriptional regulation between sugar metabolism signaling and the 3′UTR of *RhBRC1* still remains unknown. The regulatory regions within the 3′UTR can influence mRNA polyadenylation, translation efficiency, localization, and stability [84]. RNA-binding proteins that can bind to those *cis*-elements are key players in the control of mRNA stability, translation, and localization [43]. In addition, the functional characterization of RNA-binding proteins has showed that these proteins possess several conserved motifs and domains such as RNA-recognition motifs (RRMs), zinc fingers, K homology (KH) domains, DEAD/DEAH boxes (highly conserved (Asp-Glu-Ala-Asp) motifs in RNA helicases), pentatricopeptide-repeat (PPR) domains, and PUF domains [42]. Among the domains mentioned above, the PUF protein can bind *cis*-elements that contain a UGUR (R: purine) motif [85,86]. Sucrose-supplied in-vitro-cultured buds exhibited a high ability to grow out, coupled with downregulation of *RhBRC1* [27] and upregulation of *RhPUF4* (Figure 5). At the plant scale, *RhPUF4* was more abundant in non-dormant buds than in dormant ones. This sucrose-mediated *RhPUF4* was tightly linked to the OPPP in buds (Figure 6D–F) as well as in calluses (Figure S5). These findings support that the *RhPUF4* level is more likely controlled by an OPPP signal, and is stimulated when the OPPP is active and buds can grow out. It is thus tempting to speculate that RhPUF4 may act as a mediator between the OPPP and the 3′UTR of *RhBRC1* (Figure 8). This is supported by the fact that *RhPUF4* is closely related to *APUM2* (Figure 4A), which has a high binding affinity to a conserved sequence containing a core motif of UGUR flanked by an NRKR motif [49,50]. Moreover, that motif also exists in the 3′UTR of *RhBRC1* (Figure 1A). In that condition, OPPP-mediated upregulation of *RhPUF4* can reduce mRNA stability and/or translation of *RhBRC1* by binding to its own 3′UTR. 

While the transcription level of *RhPUF4* is regulated by the OPPP, it might not be regulated by glycolysis. Buds treated with sucrose + 2-DOG, glycerol, or pyruvate exhibited no significant modification of *RhPUF4* levels. It seems that *RhPUF4* may not be mediated by glycolysis/TCA-cycle-dependent signals, and this arises the question of the involvement of another posttranscriptional player. One possible candidate could be a microRNA; in *Homo sapiens* some microRNAs are regulated by glycolysis and regulate their target genes [87,88].

Our findings highlight a new mechanistic link between sugar availability and the regulation of BRC1, a major hub of shoot branching regulation, and pave the way for investigating the prevalence of this regulation in interaction with the main shoot-branching-related hormones. 

## 4. Materials and Methods

### 4.1. Cloning and Transformation

To isolate the 3′UTR of *RhBRC1* (207 bp), genomic DNA was extracted from leaves of a *Rosa hybrida* knock out mutant using a NucleoSpin Plant II kit (Machery-Nagel Inc., Düren, Germany). A primer pair (Pr3′UTRs: 5′-CACCTAACACCGCGATGAATATCGATC-3′ and Pr3′UTRas: 5′-AATGAGAAAGGTGGAAATTAGGTAG-3′) was designed to amplify the 3′UTR sequence. The 4-base-pair sequence (CACC) required for directional cloning in pENTR was added on the 5′ end of the forward primer. To isolate the NOS-terminator-modified sequence, a 256-bp sequence was designed with two modifications within NOS terminator: G103C and G117C. The sequence was cloned into a pEx-A2 vector provided by EUROFINS (Luxembourg, Luxembourg). A primer pair (NOS-Ts: 5′-CACCTAAGATCGTTCAAACATTTGGCA-3′ and NOS-Tas: 5′-GATCTAGTAACATAGATGACACCGC-3′) was designed to amplify the sequence from the modified pEx-A2-Nos-T. The 4-base-pair sequence (CACC) required for directional cloning in pENTR was added on the 5′ end of the forward primer. To isolate CDS of *APUM2* (2033 bp), total *Arabidopsis* RNAs were extracted from two-week-old *Arabidopsis thaliana* Col-0 seedlings using an RNeasy Plant Mini Kit (Qiagen, Hilden, Germany) according to the manufacturer’s recommendations. cDNA was obtained by reverse transcription performed on 1 μg of RNA using SuperScript III Reverse Transcriptase (Invitrogen, Carlsbad, USA). cDNAs from two-week-old *Arabidopsis thaliana* Col-0 seedlings were used as templates to amplify *APUM2* with a specific primer pair (APUM2_F: 5′-CACCATGATACCGGAACTGGGGAG-3′ and APUM2_R: 5′-TTACGCCATCTGAGGCTGG-3′). PCR amplifications were carried out by initial denaturation at 98 °C for 30 s followed by 35 cycles of 98 °C denaturation for 30 s, 60 °C annealing for 30 s, and 72 °C elongation for 2 min, with a final extension step at 72 °C for 10 min. The 20-μL reaction mixture for the PCR consisted of an aliquot of 35 ng of DNA template, 0.2 mM of each dNTP, 0.4 unit of Phusion DNA polymerase, and 10 pmol of each of the primers. The PCR products were separated in 1% (*w*/*v*) agarose gel and purified using a Wizard SV Gel and a PCR Clean-Up System kit (Promega, Madison, USA).

The PCR products of the *RhBRC1* 3′UTR, T’NOS, and *APUM2* were sub-cloned into an entry vector using a pENTR Directional TOPO Cloning Kit (Invitrogen). The ligation products were transferred into One Shot TOP10 Competent *E. coli* by thermal shock at 42 °C. The plasmids of several bacterial clones were extracted using a NucleoSpin plasmid extraction kit (Macherey-Nagel, Germany), and confirmed by sequencing using two different primers (M13F: 5′-GTAAAACGACGGCCAG-3′ and M13R: 5′-CAGGAAACAGCTATGAC-3′). From positive entry vectors, the three sequences were then cloned into the pGWB6 destination vector [92], using an LR Clonase II kit (Invitrogen). The ligation products were transferred into One Shot TOP10 Competent *E. coli* by thermal shock at 42 °C.

### 4.2. Rosa Callus and Arabidopsis Transformation

In vitro-propagated shoots of *Rosa* were used as starting material. They were repeatedly sub-cultured every 6 weeks on shoot multiplication medium [93] consisting of Murashige and Skoog [MS] salts and vitamins with 0.1 g·L^−1^ Fe-EDDHA, 30 g·L^−1^ sucrose, 0.1 g·L^−1^ myo-inositol, 4.44 μM 6-benzyladenine, solidified by 3 g·L^−1^ Phytagel. Young leaves were injured by several cuts and inoculated with *Agrobacterium* (EHA105) suspended in re-suspension medium until DO_600_ = 1 for 5 min. The inoculated leaves were blot-dried on sterile paper and transferred to the callus induction medium [94] completed with cefotaxime (500 mg·L^−1^) and kanamycin (100 mg·L^−1^). Leaf discs were sub-cultured every 6 weeks on the same medium until the calluses formed. Genomic DNA was extracted from the selected calluses, and PCR was used to confirm that the target fragment and GFP sequence had been stably transformed into the calluses. *Arabidopsis thaliana* wild-type Col-0 ecotype calluses were transformed by the floral dip method, using *Agrobacterium tumefaciens* carrying the P35S:GFP::APUM2 construct [95]. *apum2* mutant seeds (Salk_057880) were obtained from the NASC Institute.

### 4.3. Callus Treatment and GFP Quantification 

The transformed calluses were placed in liquid basic medium (Murashige and Skoog [MS] containing salts and vitamins) containing the different treatments for 8 h at 22 °C under light. GFP intensity was assessed under a fluorescence microscope, and quantification was performed on 2D images using ImageJ software [96]. The integrated density of gray values was determined on 30 randomly selected spots on a representative part of each sample. Each modality was replicated three to eight times.

### 4.4. Plant Culture and In-Vitro Cultivation of Axillary Buds 

For the experiments on *Rosa hybrida*, cuttings from cloned mother plants were grown in a greenhouse where the temperature was maintained around 22 °C. Nodes from the median part of the stem were harvested on single-axis plants when the floral bud was visible (VFB stage), as previously described [18,23,27]. For decapitated plants, the stems of plants with a terminal floral bud (0.5 cm above the fifth basal five-leaflet leaf) were removed. After 24 h, the lateral buds from the third and fourth basal five-leaflet leaf were collected for qRT-PCR. For in-vitro-cultured buds, 1.5-cm stem segments were transferred in vitro on classical solid MS medium with different sucrose metabolism effectors (2-DOG for glycolysis, 6-AN for the OPPP) or different products of the sucrose metabolism pathway, in a growth chamber (Strader) with 16 h light, at a temperature of 23/20 °C (day/night).

### 4.5. Rosa RNA Extraction

Total RNAs were extracted from the in-vitro-cultured buds using an RNA NucleoSpin kit (Macherey-Nagel) [27]. The absence of genomic DNA contamination was checked by PCR using specific primers designed against an intron region of the *RhGAPDH* gene [18,19]. cDNA was obtained by reverse transcription performed on 1 μg of RNA using SuperScript III Reverse Transcriptase (Invitrogen, Inc).

### 4.6. qRT-PCR

Quantitative real-time PCR (qRT-PCR) was performed with SYBR Green Supermix (Bio-Rad, Inc, Hercules, USA) using cDNA as a template, with the following program: 30 s at 98 °C, and then 40 cycles of 15 s at 95 °C and 30 s at 55 °C. Specific pairs of primers were selected according to their melting curves. Fluorescence detection was performed using a CFX Connect^TM^ Real-Time System (Bio-Rad, Inc). Quantification of relative gene expression was determined using *RhUBC* expression as an internal control [97,98]. The *RhPUF4* expression level was assessed using the primers qPrRhPUF4 (Forward, 5′-GCTTGCTGCCCTGAATGAT-3′; Reverse, 5′-GCAAGGCTCCAAGATACGC-3′), and each PCR result corresponded to three biological replicates. 

### 4.7. Statistical Analyses

R software was used for statistical treatment. One-way ANOVA (α = 0.05) was run to test for the effects of different modalities on bud outgrowth, gene transcription, and fluorescence. Significant differences are indicated by different letters or asterisks directly on the figures.

## Figures and Tables

**Table 1:** Specific PCR primers of each RhPUF member.

Gene Name	Sequence	
PrRhPUF1.	Forward	5′ GAGGAACATGAGTGGAGGTCT 3′
	Reverse	5′ CATTTGAAGGCTAAGGGTCAG 3′
PrRhPUF2	Forward	5′ TGCCCTACCAGAACGGTTTA 3′
	Reverse	5′ CAGCAAGAGCCTGACAACACT 3′
PrRhPUF3	Forward	5′ ATGGCTTAGGTGGGTTTGGT 3′
	Reverse	5′ ACTGACAATGCCGTCTGGAA 3′
PrRhPUF4	Forward	5′ CTTGAAACAGCCACTACGGA 3′
	Reverse	5′ GGTCATCACAAGTCTCCAACAC 3′
PrRhPUF5	Forward	5′ TCAGGTCCTCTTCTTGTCCG 3′
	Reverse	5′ TCCCTTTCAGTGCCTTATTCC 3′
PrRhPUF6	Forward	5′ ATGCAGCACATGCTCTGG 3′
	Reverse	5′ TAAGTTGGTTCGTCAATTCGT 3′
PrRhPUF7	Forward	5′ AGCGTCCAATCATGCCACTAG 3′
	Reverse	5′ ATACTGGTCCTGAGCAAGAGCA 3′
PrRhPUF8	Forward	5′ TAGTGGCAGTTCAGGCAATC 3′
	Reverse	5′ TCCATCCGTCCCTGTTAGTC 3′
PrRhPUF9	Forward	5′ TCTTGCACTAAGATGCCAATG 3′
	Reverse	5′ CAGCTTATCTCGATGTCTCCC 3′
PrRhPUF10	Forward	5′ ATACAAAGCCATTGCCTCAG 3′
	Reverse	5′ CTTGCAGATCAATCGGTCTC 3′
PrRhPUF11	Forward	5′ TGCACAATATGGTGCGAGTG 3′
	Reverse	5′ CCTCTTTGAAAACAGACGCCT 3′
PrRhPUF12	Forward	5′ GCAGCGATAACCAGTTAGGC 3′
	Reverse	5′ TCTCTCAGCTCCAAACATATGC 3′

## Supplementary Materials

The supplementary materials are available online at https://www.mdpi.com/1422-0067/20/15/3808/s1.
