# Supplementary material for: Posttranscriptional Regulation of RhBRC1 (Rosa hybrida BRANCHED1) in Response to Sugars is Mediated via its Own 3′ Untranslated Region, with a Potential Role of RhPUF4 (Pumilio RNA-Binding Protein Family)
Source: Int J Mol Sci. 2019 Aug 4;20(15):3808. doi: 10.3390/ijms20153808 (PMC6695800; doi:10.3390/ijms20153808)
Supplement: Supplementary file 1 [file ijms-20-03808-s001.zip › Supplementary Files/Figure S1.pdf]

3’UTR of *α-amylase 3*
CGGCUCAAGCCCUAAACUGAACGGGAUAGUCAUGCUCAAACCAGUUUCUACACGGCAAGAAUUUACUGA
UUCUUAUACUUUUGCAGUCAAUUAAAUUAUGGUUUUUAUAUAUGUAAUUUUGUAUCCGAUUGUAGUUG
UAUCCGAUUGUAGCGUUCGAAUAAGUAGGCAGGCUCUCUAGCCUCUAGGUUAAUUGCGGGGCAUAUGU
AGCUUGCCAGGCAUAUGUAGCUUGCCAGUUAAUUGUGUUUGUAUCACGCAGUUUGUAACCGUUGGUGC
AAUAUAUAAUGUCAGGUUCAGG

3’UTR of *AtBRC1*
AACUGAUUUCAUUUAACAUUAAUAUCUCAAAUUAAUGAUAUAUAGUUUGGUGAGAGAGAUAUGUUAAU
CCAUCUUAUGUUUUUUUUUCUUAACAUCGUAAUUAAUCAUGUUUAUAUGAAGGCUUUUUCGUCAUUUU
AGUGUCAAAUAUUAAACCUAUGUGAAGAAAUUAAUGCAAUUUGUAUAAUAUUUUGUUUUCAUAGUUCG
AGUGGAU

3’UTR of *OsTB1*
UGUGAUCAUCCAUCCACACACGAACGAACGAACGAACGGUACGGCACUAAGAUCGAACUCCUGCAGCUA
CAUAAUUAUCCUUUGCUUCUCAAGAGUAAUAAUUCUUGACGUGUUAAUUAAUCCGGGUGUGUAUUAAU
UCCCUCUUUAUUAUUUUUUCUCGCGUUUAUCCGGAGUUGACUGUGGUGAAGACGAACUUUGGUUUGGU
CAUCGCAUGGUGUGCAUUGCAUAUAUAGCUAGCACUAUCGUCUGAUCGAUGAUUCAUCAUCAAUGGAGU
CGUCUAGUAUUAA
