# Supplementary material for: Posttranscriptional Regulation of RhBRC1 (Rosa hybrida BRANCHED1) in Response to Sugars is Mediated via its Own 3′ Untranslated Region, with a Potential Role of RhPUF4 (Pumilio RNA-Binding Protein Family)
Source: Int J Mol Sci. 2019 Aug 4;20(15):3808. doi: 10.3390/ijms20153808 (PMC6695800; doi:10.3390/ijms20153808)
Supplement: Supplementary file 1 [file ijms-20-03808-s001.zip › Supplementary Files/Figure S3.pdf]

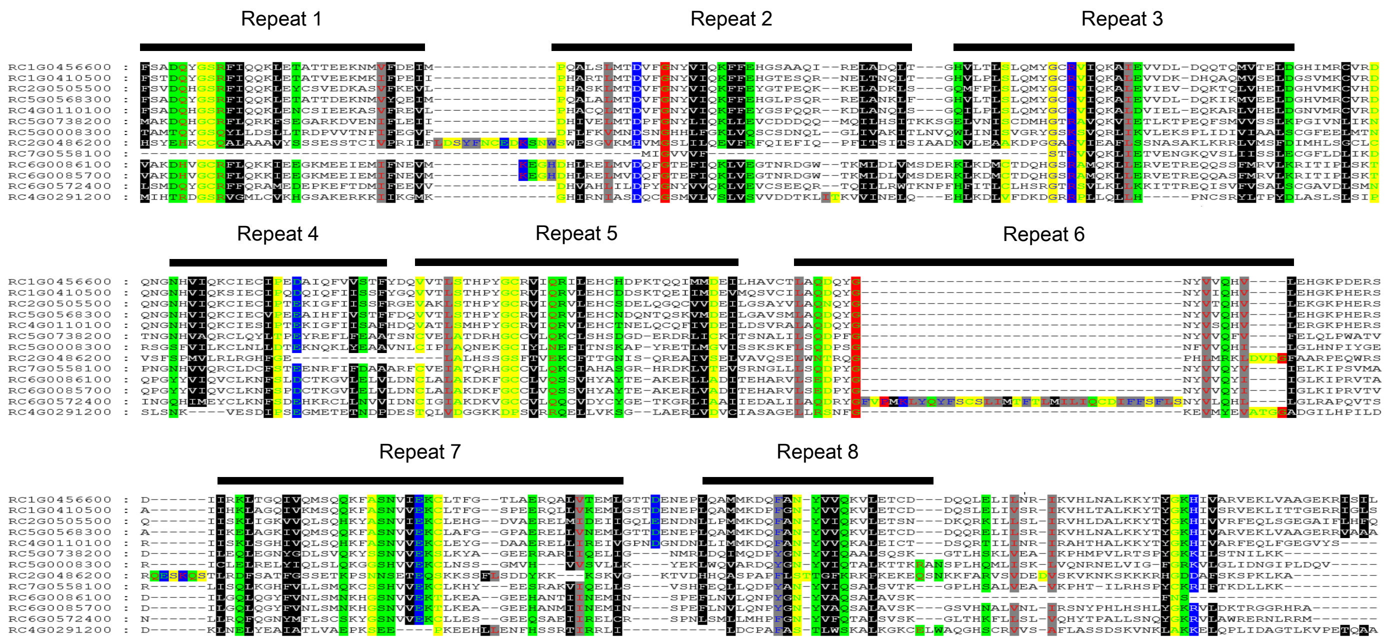
Repeat 1
Repeat 2
Repeat 3
Repeat 4
Repeat 5
Repeat 6
Repeat 7
Repeat 8
RC1G0456600
RC1G0410500
RC2G0505500
RC5G0568300
RC4G0110100
RC5G0738200
RC5G0008300
RC2G0486200
RC7G0558100
RC6G0086100
RC6G0085700
RC6G0572400
RC4G0291200
