# Supplementary material for: Posttranscriptional Regulation of RhBRC1 (Rosa hybrida BRANCHED1) in Response to Sugars is Mediated via its Own 3′ Untranslated Region, with a Potential Role of RhPUF4 (Pumilio RNA-Binding Protein Family)
Source: Int J Mol Sci. 2019 Aug 4;20(15):3808. doi: 10.3390/ijms20153808 (PMC6695800; doi:10.3390/ijms20153808)
Supplement: Supplementary file 1 [file ijms-20-03808-s001.zip › Supplementary Files/Supplementary Figures.docx]

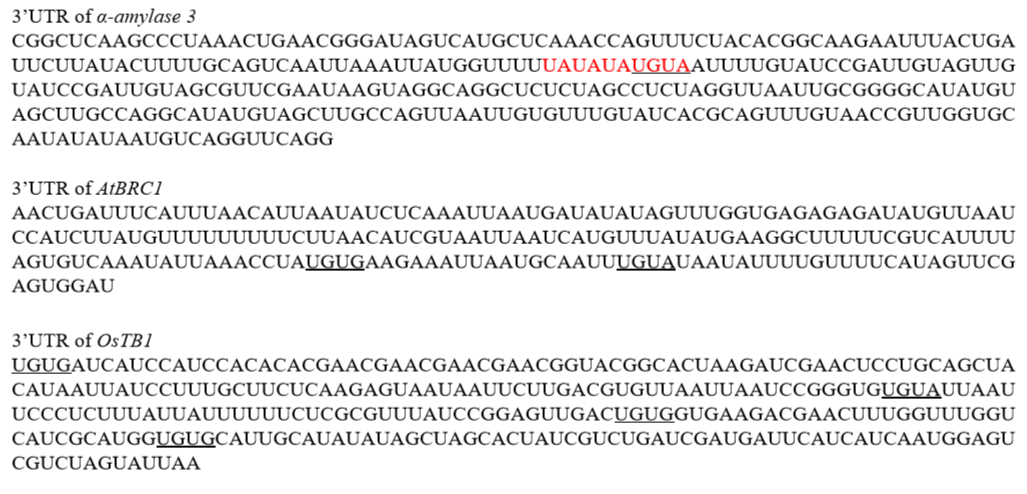


**Figure 1.** 3’UTRs of *α-amylase 3*, *AtBRC1,* and *OsTB1‎*. The 3’UTRs were retrieved from the NCBI database ([www.ncbi.nlm.nih.gov](http://www.ncbi.nlm.nih.gov)). Red letter indicates the sugar-related motif found in *Oryza sativa α-amylase 3.* Underlined letters indicate the core PUF-binding motif*.*


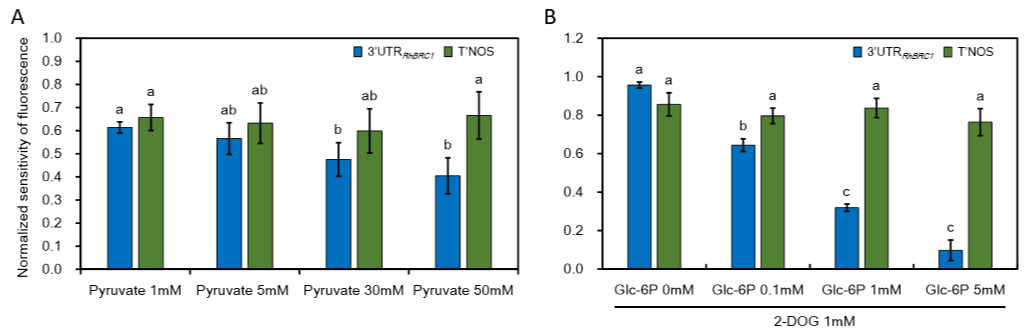


**Figure 2.** The fluorescence level of 3’UTR*_RhBRC1_*-transformed calluses (P35S:GFP::3’UTR*_RhBRC1_*) is sensitive to the OPPP, but only slightly sensitive to the glycolysis/TCA-cycle. A, 3’UTR*_RhBRC1_*-transformed calluses were treated with different pyruvate concentrations; B, 3’UTR*_RhBRC1_*-transformed calluses were treated with 1 mM 2-DOG and with different glucose-6-phosphate concentrations. Glc-6P, glucose-6-phosphate. Data are means ± SEs of three measurements, each measurement was performed on six calluses. The letters indicate significant differences between the different treatments with *P＜0.05*.


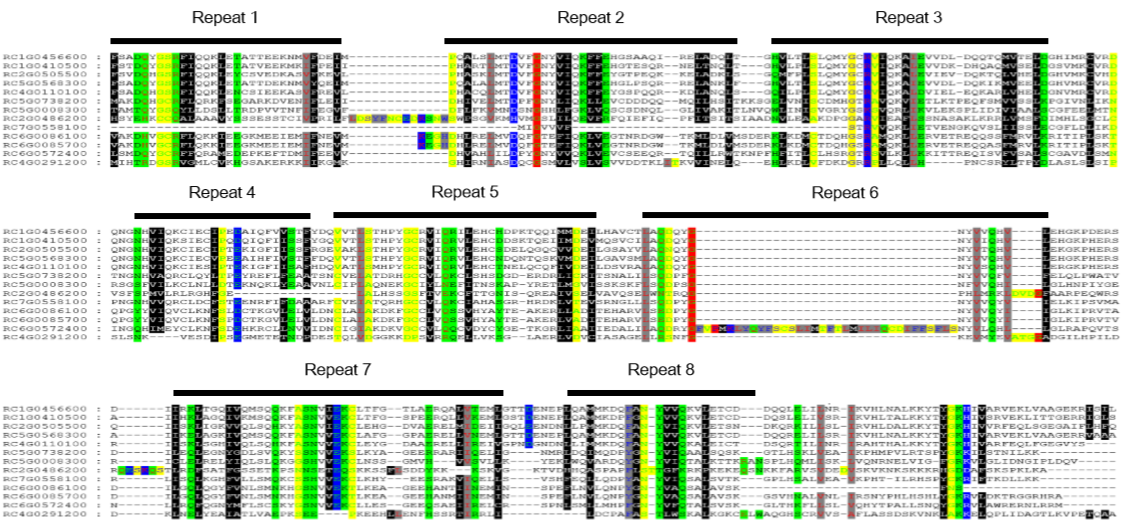


**Figure 3.** Sequence alignment of Pumilio repeats among PUF members in *Rosa chinensis*. Multiple alignments were generated using ClustalX program. The PUF sequences of *Rosa chinensis* were downloaded from the GDR database (https://www.rosaceae.org/)


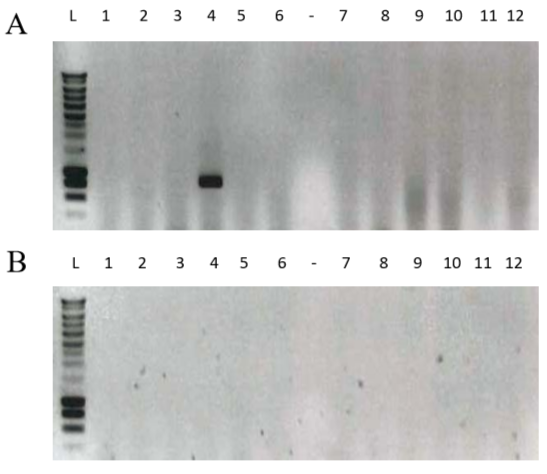


**Figure 4.** Expression levels of 12 putative *RhPUF* members in buds treated with 100 mM sucrose or 100 mM mannitol. A, Expression levels of 12 *RhPUF* members in buds treated with 100 mM sucrose. B, Expression levels of 12 *RhPUF* members in buds treated with 100 mM mannitol. L, Ladder; 1, *RhPUF1*; 2, *RhPUF2*; 3, *RhPUF3*; 4, *RhPUF4*; 5, *RhPUF5*; 6, *RhPUF6*; 7, *RhPUF7*; 8, *RhPUF8*; 9, *RhPUF9*; 10, *RhPUF10*; 11, *RhPUF11*; 12, *RhPUF12*; -, negative control.


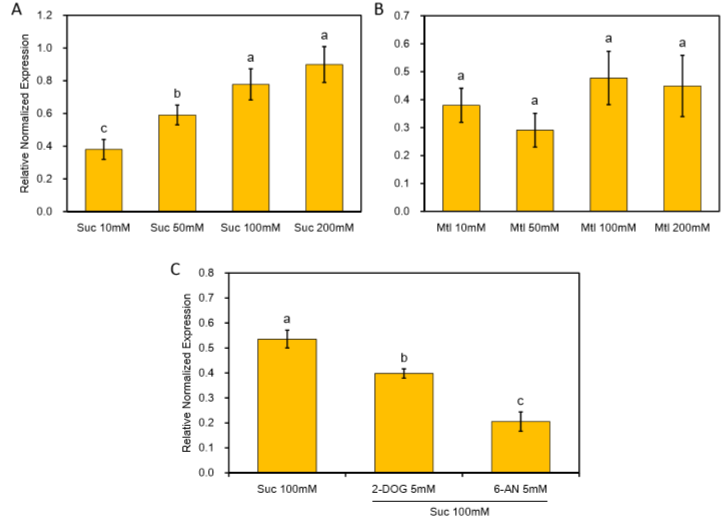


**Figure 5.** Transcription level of *RhPUF4* is regulated by sucrose concentration and sensitive to OPPP in callus. A&B, Transcript level of *RhPUF4* in callus treated with different sucrose or mannitol concentration respectively; C, Transcription level of *RhPUF4* in callus is both inhibited by 2-DOG and 6-AN. Suc, sucrose; Mtl, mannitol. Data are mean ± SE of three repetitions. The letters indicate significant differences between the different treatments with *P＜0.05*.
