# Supplementary figures and images for: Posttranscriptional Regulation of RhBRC1 (Rosa hybrida BRANCHED1) in Response to Sugars is Mediated via its Own 3′ Untranslated Region, with a Potential Role of RhPUF4 (Pumilio RNA-Binding Protein Family)
Source: Int J Mol Sci. 2019 Aug 4;20(15):3808. doi: 10.3390/ijms20153808 (PMC6695800; doi:10.3390/ijms20153808)

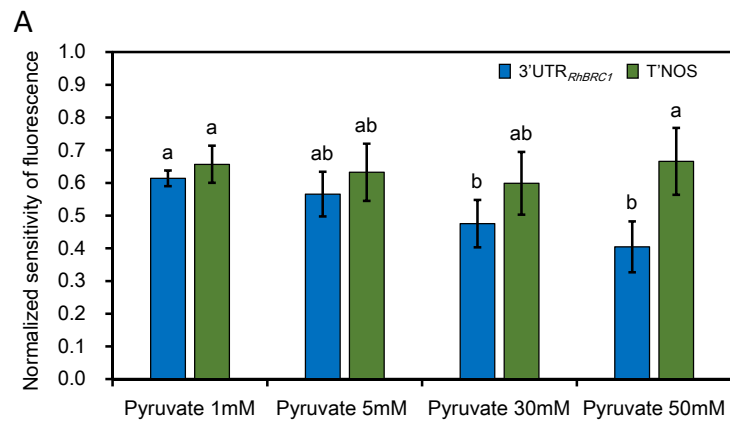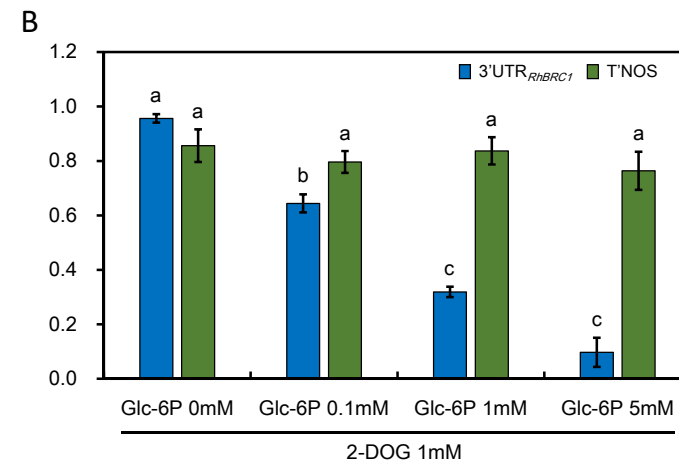

Supplement: Supplementary file 1 [file ijms-20-03808-s001.zip › Supplementary Files/Figure S2.pdf]

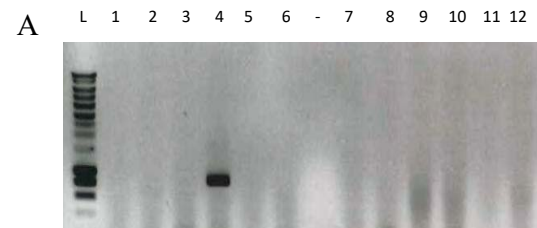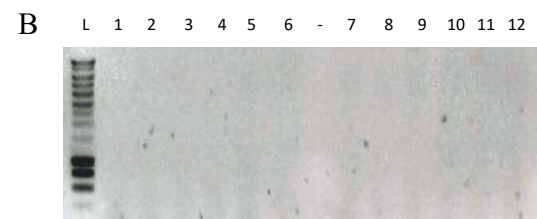

Supplement: Supplementary file 1 [file ijms-20-03808-s001.zip › Supplementary Files/Figure S4.pdf]

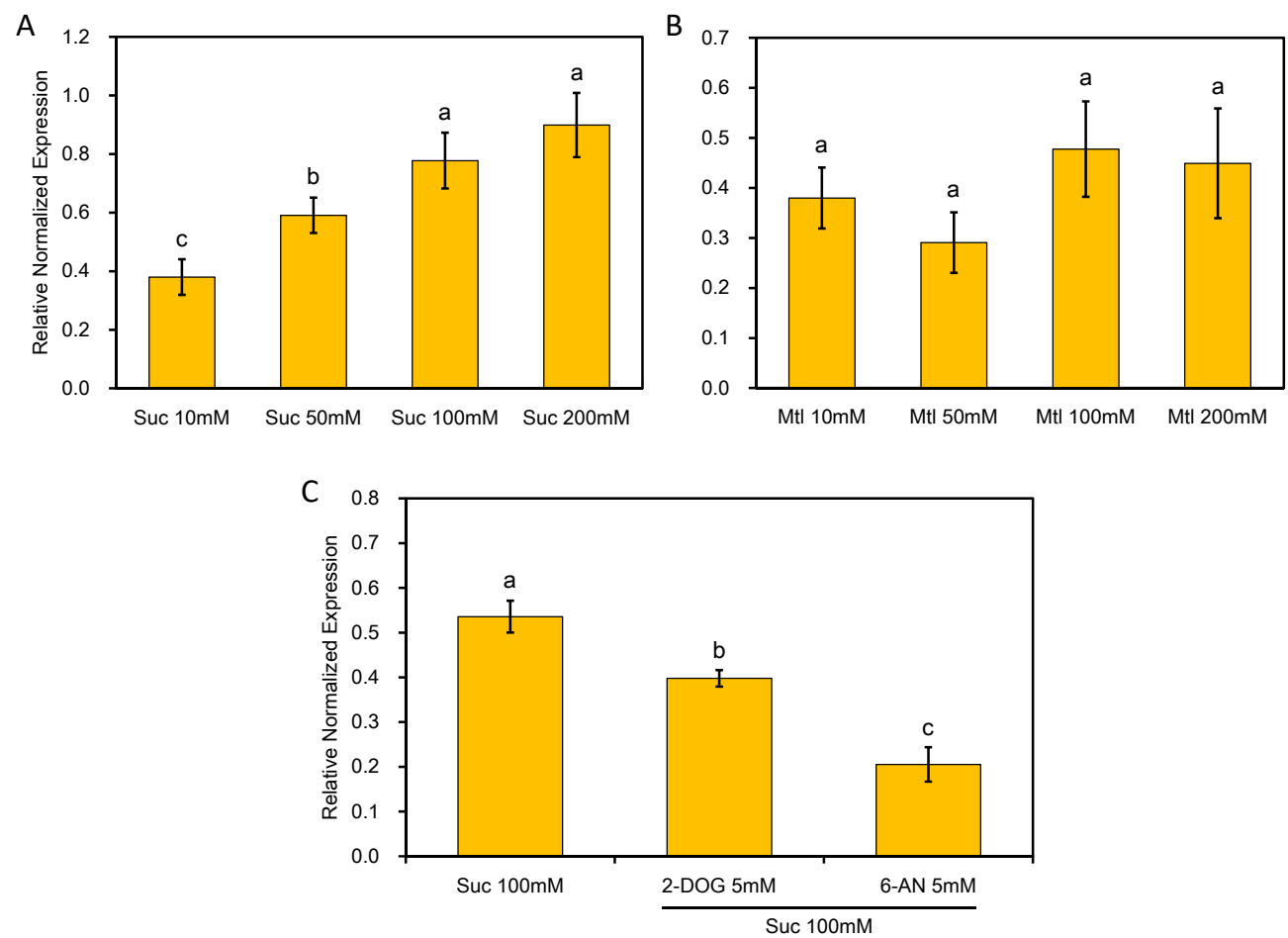

Supplement: Supplementary file 1 [file ijms-20-03808-s001.zip › Supplementary Files/Figure S5.pdf]
